# Expanding the RNA polymerase biocatalyst solution space for mRNA manufacture

**DOI:** 10.1002/biot.202400012

**Published:** 2024-06-21

**Authors:** Edward Curry, Svetlana Sedelnikova, John Rafferty, Martyn Hulley, Melinda Pohle, George Muir, Adam Brown

**Affiliations:** ^1^ Department of Chemical and Biological Engineering University of Sheffield Sheffield UK; ^2^ School of Biosciences University of Sheffield Sheffield UK; ^3^ Bioprocess Development AstraZeneca Cambridge UK

**Keywords:** cell‐free screening, in vitro transcription, mRNA manufacture, RNA polymerase

## Abstract

All mRNA products are currently manufactured in in vitro transcription (IVT) reactions that utilize single‐subunit RNA polymerase (RNAP) biocatalysts. Although it is known that discrete polymerases exhibit highly variable bioproduction phenotypes, including different relative processivity rates and impurity generation profiles, only a handful of enzymes are generally available for mRNA biosynthesis. This limited RNAP toolbox restricts strategies to design and troubleshoot new mRNA manufacturing processes, which is particularly undesirable given the continuing diversification of mRNA product lines toward larger and more complex molecules. Herein, we describe development of a high‐throughput RNAP screening platform, comprising complementary in silico and in vitro testing modules, that enables functional characterization of large enzyme libraries. Utilizing this system, we identified eight novel sequence‐diverse RNAPs, with associated active cognate promoters, and subsequently validated their performance as recombinant enzymes in IVT‐based mRNA production processes. By increasing the number of available characterized functional RNAPs by more than 130% and providing a platform to rapidly identify further potentially useful enzymes, this work significantly expands the RNAP biocatalyst solution space for mRNA manufacture, thereby enhancing the capability for application‐specific and molecule‐specific optimization of both product yield and quality.

## Graphical Abstract and Lay Summary

RNA polymerases (RNAPs) are used to produce mRNA for therapeutic purposes, however the toolbox of available RNAPs is limited. In this work, a high throughput cell free screening system is introduced, that allows for the rapid characterization of putative RNAPs. 8 novel RNAPs are described, in addition to providing a means by which new or engineered RNAPs can be rapidly assessed for their suitability for mRNA manufacture.

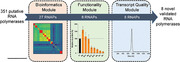

**Abbreviations**
CGE: capillary gel electrophoresis
CLuc: *Cypridina* luciferase
dsRNA: double‐stranded RNA
IVT: in vitro transcription
RNAP: RNA polymerase

## 1 INTRODUCTION

The clinical success of SARS‐Cov‐2 vaccines established synthetic mRNA as an effective drug format, paving the way for hundreds of new mRNA‐based vaccines and gene therapies to enter clinical trials.^[^ ^1^, ^2^ ^]^ This has resulted in a sharp increase in global demand for mRNA production, and shifted mRNA manufacturing from a relatively niche process to one that underpins current and future strategies to treat monogenic disorders, cancer, and infectious diseases.^[^ ^3^, ^4^, ^5^ ^]^ All such mRNA products are currently produced in standardized in vitro transcription (IVT) systems using single‐subunit DNA‐dependent phage RNA polymerase (RNAP) biocatalysts. While *Salmonella* phage SP6 and *Enterobacteria* phage T3 RNAPs can be utilized in certain contexts, the dominant biocatalyst choice for synthetic mRNA manufacture is the *Enterobacteria* phage T7 RNAP. This enzyme has undergone extensive protein engineering to improve bioproduction performance, predominantly via strategies to reduce formation of immunogenic product‐related impurities such as short‐abortive transcripts^[^ ^6^, ^7^ ^]^ and double‐stranded RNA (dsRNA) species.^[^ ^8^, ^9^ ^]^

Although T7 RNAP typically generates high product yields and acceptable product quality profiles, it is highly unlikely that a single one‐size‐fits‐all biocatalyst approach will be optimal for all mRNA manufacturing contexts. Indeed, other bioproduction processes rely on biocatalyst toolbox approaches, such as the wide range of evolved and engineered Chinese Hamster Ovary cell factories utilized for recombinant protein manufacture.^[^ ^10^ ^]^ The current unavailability of such an RNAP toolbox for mRNA IVT platforms restricts (i) bioprocess design strategies, such as optimizing temperature set‐point to achieve quality target product profiles, and (ii) molecule/application specific optimization of product yield and quality. The latter is particularly pertinent given that mRNA product lines are diversifying to include larger and more complex molecular formats that present new biomanufacturing challenges, such as circular RNA,^[^ ^11^, ^12^, ^13^ ^]^ long self‐amplifying RNA transcripts,^[^ ^14^, ^15^ ^]^ and linear mRNA incorporating novel cap structures and modified nucleotides.^[^ ^16^ ^]^ Indeed, it should be anticipated that mRNA will follow the path of protein therapeutics, where product designers rapidly progressed from relatively simple molecules such as insulin to highly engineered formats (e.g., tri‐specific antibodies) that require product‐specific biocatalyst solutions.^[^ ^17^ ^]^

The available RNAP toolbox has recently been expanded by studies focused on identifying and characterizing individual enzymes with putative desirable bioproduction phenotypes. KP34 enhances 3′ homogeneity of product molecules by ≈40‐fold compared to T7, a result of being unable to catalyze self‐templated 3′ RNA end extension.^[^ ^18^ ^]^ Syn5 exhibits unusually high levels of processivity and can produce larger transcripts at higher quantities and quality than T7, owing to its natural function of producing extended polycistronic mRNAs.^[^ ^19^ ^]^ VSW‐3 reduces dsRNA impurities by ≈100‐fold compared to T7, due to lacking any detectable RNA‐dependent RNAP activity.^[^ ^20^ ^]^ While these hypothesis‐driven approaches have successfully identified new biocatalysts with novel functionalities, only six characterized RNAPs are currently publicly available for mRNA manufacture (although we note that some additional unpublished enzymes may be utilized in industrial settings). Accordingly, mRNA manufacturing solution spaces are severely limited by the restricted number of RNAPs available for bioproduction. Moreover, currently utilized “standardized” biocatalysts such as T7 may have relatively poor performance characteristics relative to the hundreds of “untested” single‐subunit phage RNAPs found in nature, including lower processivity^[^ ^19^ ^]^ and higher generation rates of impurities such as dsRNA^[^ ^21^ ^]^ and abortive transcripts.^[^ ^6^ ^]^

In this study, we address the paucity of biocatalysts available for IVT‐based mRNA production. Using a combination of in silico and in vitro analyses we identify and functionally validate eight new sequence‐diverse RNAPs, more than doubling the number of previously described enzymes for mRNA manufacture. In doing so, we describe development of a high‐throughput screening system that can be utilized to rapidly select and test future RNAP libraries, facilitating further expansion of the biocatalyst solution space. Provision of a substantially expanded RNAP toolkit enhances capabilities to design and troubleshoot new molecule‐specific manufacturing processes, which will be particularly useful for optimizing yield and quality of complex next‐generation mRNA products.

## 2 MATERIALS AND METHODS

### 2.1 RNAP library creation

A starting library of 351 predicted RNAP sequences was collated from Uniprot, comprising all sequences annotated as predicted phage DNA‐directed RNAPs. RNAPs were clustered by grouping RNAPs sharing sequence identity >85%, using the Clustal Omega online alignment tool.^[^ ^22^ ^]^ RNAPs were further clustered using the MMSEQ2 online server, with a minimum sequence identity threshold of 85%, and coverage threshold of 70%.^[^ ^23^ ^]^ A representative RNAP from each cluster was chosen by totaling the matrix identity score to determine which polymerase in each cluster was most divergent in sequence to all others. Promoters for remaining RNAPs were predicted by PHIRE (Phage in silico regulatory elements)^[^ ^24^ ^]^ using parameters of string lengths – 20, window size – 30, and degeneracy – 4. Predicted promoter sequences were verified with the *PhagePromoter* tool,^[^ ^25^ ^]^ with a probability threshold of 0.5.

### 2.2 Plasmid construction

For coupled transcription‐translation assay plasmids, RNAP sequences were synthesized and cloned into XhoI and XbaI restriction sites on the pTNT vector (Promega). To create the corresponding transcription templates, the NanoLuc gene (Promega) was cloned into XhoI and XbaI restriction sites in pTNT, before site directed mutagenesis to substitute the SP6 and T7 promoter with the promoter of interest. For RNAP overexpression plasmids, RNAP sequences were inserted between NdeI and XhoI sites on pET‐29b (Novagen). Transcription templates were made by site directed mutagenesis to substitute the T7 promoter with the promoter of interest on pCMV‐Cluc2 (New England Biolabs).

### 2.3 Cell free coupled transcription‐translation assays

Coupled transcription‐translation assays were carried out using the TNT SP6 Quick Coupled transcription/translation system (Promega). Reactions were assembled containing 8 µL TnT Quick Master Mix, 1 µL RNAP plasmid (40 ng µL^−1^), 1 µL NanoLuc plasmid (80 ng µL^−1^), and 0.2 µL 1 mm methionine. The assay proceeded at 30°C for 1 h. Samples were then diluted 500‐fold in nuclease free water, and added at a 1:1 ratio to pre‐diluted NanoLuc luciferase assay substrate. Samples were incubated in darkness for 5 min, before detection of luminescence by Molecular Devices ID5 plate reader, with an integration time of 10 s.

### 2.4 Cell free coupled transcription‐translation for quality assessment

To produce mRNA for quality assessment, coupled transcription‐translation assays were carried out using the TNT SP6 Quick Coupled transcription/translation system (Promega). Reactions were assembled containing 64 µL TnT Quick Master Mix, 1 µL RNAP plasmid (400 ng µL^−1^), 1 µL NanoLuc plasmid (400 ng µL^−1^), and 1.6 µL 1 mm methionine, and 12.4 µL ddH_2_O. The assay proceeded at 30°C for 2 h. Polyadenylated mRNA was subsequently purified from the reaction by Dynabeads Oligo (dT)_25_ (ThermoFisher) magnetic bead based affinity enrichment, following the manufacturers protocol. Briefly, 2 mg oligo (dT)_25_ beads are equilibrated in binding buffer (20 mm Tris‐HCl, pH 7.5, 1.0 m LiCl, 2 mm EDTA), before addition of mRNA containing samples. Beads were washed twice with washing buffer (10 mm Tris‐HCl, pH 7.5, 0.15 m LiCl, 1 mm EDTA), before elution of bound poly(A) mRNA with ddH_2_O.

### 2.5 Capillary gel electrophoresis (CGE)

CGE analysis of RNA was performed with a 5200 Fragment Analyzer System (Agilent), using the DNF‐471 RNA Kit (15 nt) (Agilent). The capillary cassette used was FA 12‐Capillary Array Short, 33 cm (Agilent). Samples were diluted to <100 ng µL^−1^ in nuclease free water. Before each separation, a pre‐run voltage was applied (8 kV for 30 s), the capillaries were conditioned with the conditioning solution and the capillaries were dipped twice in the rinse buffer. Following this, the capillaries were filled with RNA separation gel (by pressure) and then the sample was introduced using a voltage injection (5 kV for 4 s). The separation was then conducted by applying a voltage of 8 kV for 45 min. Detection was carried out using laser induced fluorescence, by fluorescent dye tagging of the RNA.

### 2.6 Transfection of NanoLuc mRNA into HEK293 cells

Suspension adapted Expi293f HEK cells (Thermo Fisher) were routinely cultured in Gibco FreeStyle 293 Expression Medium (Thermo Fisher). Cells were maintained in 30 mL volume in 125 mL Erlenmeyer flasks (Corning), at 37°C, 80% humidity, and 5% CO_2_, with agitation at 140 rpm. Cell density and viability was determined by the Vicell Blue automated cell counter system (Beckman Coulter). Cells for transfection were cultured in 24‐shallow well plates (Corning), containing 500 µL culture volume, with agitation at 240 rpm. For mRNA transfection, cells were seeded at a density of 0.5 × 10^6^ cells per mL in 24‐shallow well plates, and incubated for 24 h. TransIT‐mRNA transfection reagent (Mirus) was used to transfect 3 µL of mRNA per well as per manufacturer's instructions.

### 2.7 RNAP expression and purification

pET‐29b‐RNAP plasmids were transformed into BL21 or NEB Shuffle *Escherichia coli* cells (New England Biolabs), and grown in 5 mL culture overnight at 37°C. Starter cultures were used to inoculate 500 mL LB broth, which was incubated at 37°C until an OD_600_ of 0.4 was reached. At this point incubation temperature was lowered to 25°C. Protein expression was then induced by addition of 1 m IPTG (Sigma–Aldrich), before harvesting of cells after 8 h. Cell pellets were resuspended for purification in buffer A (50 mm Tris‐HCl, 0.5 m NaCl, pH 8.0), and lysed by sonication. After removal of cell debris by centrifugation (73,000 × *g* for 15 min), cell free extract was applied to a 5 mL HisTrap HP column (Cytivia) at a flow rate of 5 mL min^−1^. The column was washed with 2 column volumes Buffer A + 40 mm imidazole (Sigma–Aldrich), before elution of protein in a gradient of imidazole from 0 to 300 mm over 10 column volumes. 5 mL of eluted protein was applied to a 1.6 × 60 cm Superdex200 gel filtration column at 1.5 mL min^−1^, with 2 mL fractions collected after void volume. Fractions containing the RNAP of interest were concentrated to a final concentration of 1 mg mL^−1^ and exchanged into RNAP storage buffer (50 mm Tris‐HCl, 100 mm NaCl, 50% v/v glycerol, 10 mm DTT, 0.1 mm EDTA, 0.2% w/v NaN3 [All Sigma–Aldrich]), using a 50 kDA MWCO centrifugal filter (Sigma–Aldrich). RNAP preparations were stored at −20°C. Purity of final preparations was assessed by Tris‐Glycine SDS‐PAGE 4%–12% BT Novex gel with MES running buffer (Invitrogen).

### 2.8 In vitro transcription

Plasmid templates for IVT were linearized with XbaI, and purified by ethanol precipitation. Transcription reactions using the Hiscribe IVT kit (New England Biolabs) were assembled to a final volume of 20 µL. Reactions contained 2 µL 10X reaction buffer, 2 µL of each NTP, 1 µg of template DNA, and 2 µL of T7 RNAP, or 2 µL of novel RNAP. Transcription reactions were incubated for 2 h, before addition of 1 µL DNase I, and further incubation for 20 min. Transcription reactions were purified using the Monarch RNA cleanup kit (New England Biolabs). mRNA concentration was quantified by nanodrop spectrophotometer, and product integrity assessed by agarose gel electrophoresis.

## 3 RESULTS AND DISCUSSION

### 3.1 Bioinformatic analysis of the potential RNA polymerase biocatalyst solution space

**FIGURE 1:**
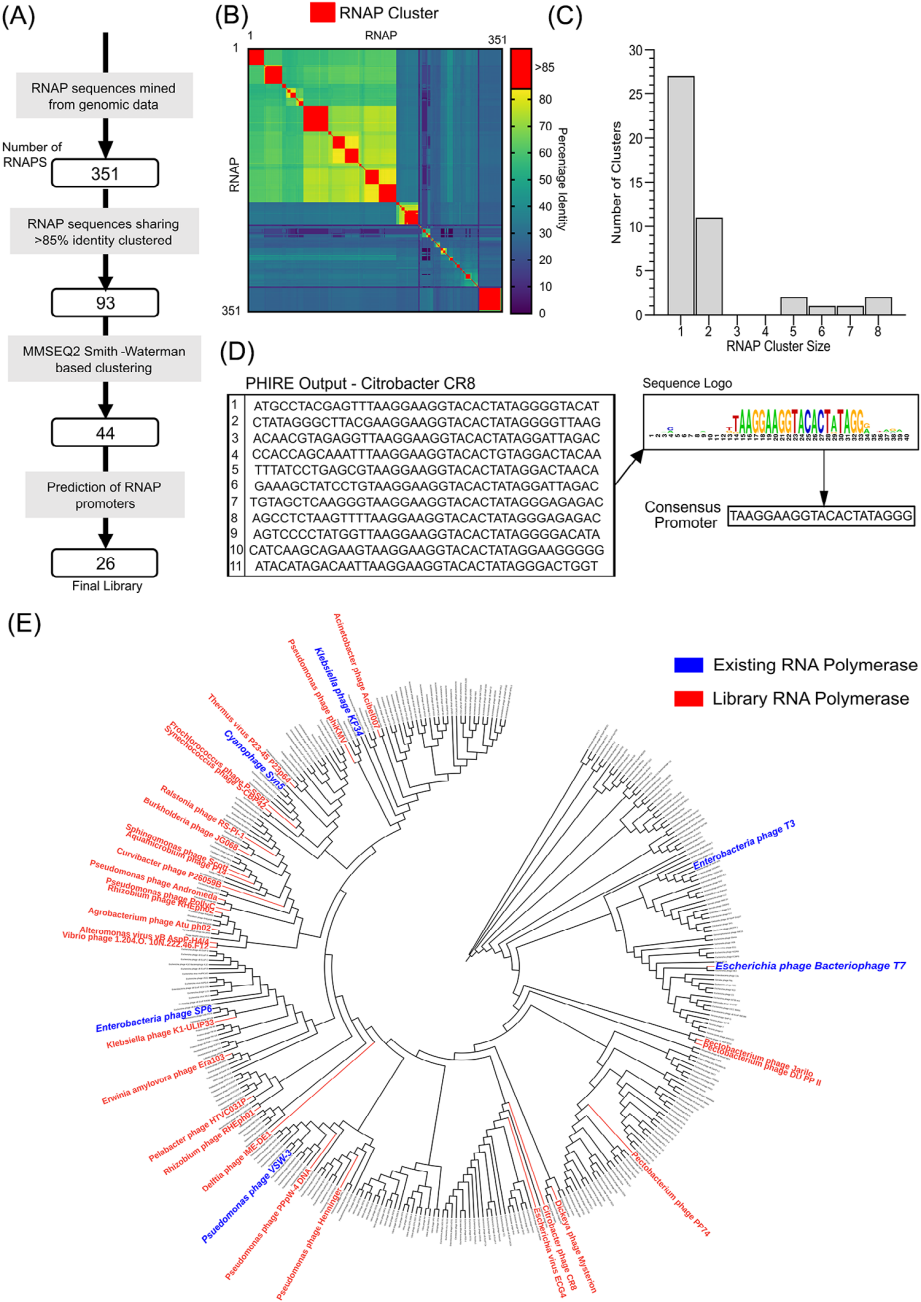
Bioinformatics‐driven design of an RNA polymerase (RNAP) “test” library (A). Putative RNAPs were clustered using pairwise global sequence identity analysis (B), and subsequently grouped into 44 distinct families according to local sequence similarities (C). Representative polymerases from families for which accurate cognate promoter prediction was possible (D) were phylogenetically analyzed to validate evolutionary diversity, represented in the circular cladogram showing all 351 analyzed RNAPs (E).

The biocatalyst solution space for mRNA production is currently limited to a handful of characterized RNAPs. To define the theoretical solution space, we extracted the sequence of all putative single subunit RNAPs from Uniprot. At the time of conducting this analysis, 351 distinct single‐stranded RNAPs had been predicted from publicly available genomics data. Accordingly, given that only six of these enzymes had been previously tested, ≈98% (i.e., 345 out of the potential 351 enzymes) of the potential solution space remained unexplored. We concluded that testing all 345 previously untested RNAPs would be inefficient, and, moreover, unnecessary, given that many of these enzymes will share similar performance characteristics. Indeed, we assumed that variation in bioproduction phenotype (e.g., enzyme processivity, impurity generation profiles) would be underpinned by significant differences in amino acid sequences. Accordingly, we sought to define distinct spots within the potential solution space by identifying RNAP clusters that shared minimal amino acid sequence homology (Figure 1A).

The Clustal Omega sequence alignment tool^[^ ^22^ ^]^ was used to define RNAP clusters, whereby enzymes with >85% global sequence identity were grouped into a single distinct family. This analysis identified 93 enzyme clusters, where the smallest and largest groups contained 1 and 32 RNAPs, respectively (Figure 1B). To interrogate local sequence similarities, these families were then analyzed using MMSEQ2, grouping RNAPs based on k‐mer matching and the Smith–Waterman algorithm. Using sequence identity and coverage thresholds of 85% and 70%, respectively,^[^ ^23^ ^]^ the number of discrete RNAP families was reduced from 93 to 44, where the majority of clusters (27) contained a single enzyme (Figure 1C).

Utilization of novel RNAPs for mRNA manufacture requires concomitant identification of appropriate cognate promoter elements to drive product transcription. This is non‐trivial as single subunit RNAPs typically display highly stringent promoter binding activity, where mutation of a single nucleotide can abolish transcriptional output.^[^ ^26^ ^]^ Indeed, the high specificity binding activity of single subunit RNAPs to promoters is highlighted by recent work showing that any changes to the T7 promoter sequence results in substantial reductions in mRNA production efficiency.^[^ ^27^, ^28^, ^29^ ^]^ Accordingly, identification of novel functional polymerases necessitates highly accurate promoter predictions. However, there are only limited publicly available tools to achieve this, where PHIRE^[^ ^24^ ^]^ searches for conserved elements of defined length, and PhagePromoter utilizes machine learning models to classify specified phage sequences as “promoter” or “non‐promoter”.^[^ ^25^ ^]^ A representative polymerase was selected from each of the 44 families, and the associated phage genomes were investigated with both of these promoter prediction tools (Figure 1D). This analysis failed to accurately identify cognate promoters in 18/44 cases. Testing further RNAPs from these 18 families similarly failed to result in identification of useable elements, indicating that the amino acid sequence diversity within these clusters is associated with “unusual” cognate binding motifs that are significantly different to the promoter datasets that were used to train existing prediction tools. Accordingly, ≈40% of identified RNAP clusters could not be tested in vitro due to limitations in promoter prediction capabilities.

Cognate promoters were successfully predicted for the remaining 26 RNAPs, and optimal reaction temperatures were identified for each enzyme based on the growth temperature of corresponding phage hosts (Table 1). Ten RNAPs had predicted temperature optima ≤30°C, which may be beneficial for mRNA product quality profiles given that IVT reactions performed at reduced temperatures are associated with decreased levels of product‐related impurities.^[^ ^20^ ^]^ Phylogenetic analysis of the 26 selected RNAPs confirmed that the final library comprised a panel of evolutionarily diverse enzymes, sharing no significant sequence similarity (≤75% global sequence identity) with any the six previously characterized polymerases (Figure 1E). These polymerase‐promoter pairs (Table 1) were taken forward for in vitro functional characterization, facilitating testing of ≈60% of the identified RNAP clusters.

**TABLE 1:** Bioinformatically identified single‐subunit RNA polymerases selected for in vitro functional characterization.

Phage name	Assigned number	Temperature optimum [°C]	Uniprot protein ID	Promoter prediction
*Prochlorococcus* phage P‐SSP7	R1	14–26	Q58N45	AAAATTCTTCAAGTTTACAA
*Synechococcus* phage S‐CBP42	R2	20	A0A096VKW2	CACTTCCACTCAACCAACCG
Erwinia amylovora phage Era103	R3	28	A2I7X6	AATAACCACCCAGTATAGAAGGAA
*Agrobacterium* phage Atu ph02	R4	28	A0A223VZI2	TTATCCTTCGTATAAGGAATA
*Dickeya* phage Mysterion	R5	28	A0A385IGY0	CTTAAATCATCACTATTAG
*Pectobacterium* phage PP74	R6	30	A0A1J0MEG1	TAATACGACTCACTATTGGGAA
*Aquamicrobium* phage P14	R7	30	A0A1L5C074	TTTCGGTACGCTCTAGCA
*Pectobacterium* phage DU PP II	R8	30	A0A2D2W5U8	TTATTAACGACTCACTACTAGGAA
*Pectobacterium* phage Jarilo	R9	30	A0A2S1GSW7	TAATAACGACTCACTATTAGAAG
*Sphingomonas* phage Scott	R10	30	A0A346FDD2	TCGGGTTGTCGATTTCCTTAC
*Ralstonia* phage RS‐PI‐1	R11	35	A0A1S6L1D6	GTCGAAGTCGTCGAGCAGC
*Burkholderia* phage JG068	R12	37	U3PFP4	TCAGTAGACTATCTAG
*Acinetobacter* Acibel007	R13	37	A0A075DXW8	CTGTACTCACAGCTCAATTT
*Delftia* phage IME‐DE1	R14	37	A0A0F7INH1	GTTAGCCCACACCATTGAAGACCC
*Pseudomonas* phage Henninger	R15	37	A0A2K9VHD7	TTAAAACCCTCACTATGGCTACA
*Pseudomonas* phage PollyC	R16	37	A0A2K9VHU7	CTCACTCACGACCCAAATTC
*Pseudomonas* phage phiKMV	R17	37	Q7Y2D9	CGACCCTTCCCTACTCCGGCCTTAAAT
*Citrobacter* phage CR8	R18	37	W6PP41	TAAGGAAGGTACACTATAGGG
*Thermus* virus P23‐45 P23p64	R19	65	A7XX94	TTATTCCTTTA
*Pelagibacter* phage HTVC031P	R20	16–23	A0A4Y1NTX3	AACTAATGCTCAATTTAGAGATA
*Rhizobium* phage RHEph01	R21	25–30	L7TQW5	ATTACCCCTCCCTTAAGCAAAG
*Rhizobium* phage RHEph02	R22	25–30	L7TJC5	TTAATCCTCACTATTAGGATAA
*Curvibacter* phage P26059B	R23	25–30	A0A384UH57	GCAACATTACAGGTACTGAA
*Pseudomonas* phage PPpW‐4	R24	25–30	V5YUU1	TAAAAACCCTCACTGAAACAGGG
*Vibrio* phage 10N	R25	35–37	A0A2I7RNL7	ACTTACCTTTCACTATAGCAGCA
*Alteromonas* virus vB AspP‐H44	R26	Not found	A0A220YL66	TGGTGACTACAGAGCAGCAG

### 3.2 Identification of novel active RNAP biocatalysts via high‐throughput in vitro functional characterization

**FIGURE 2:**
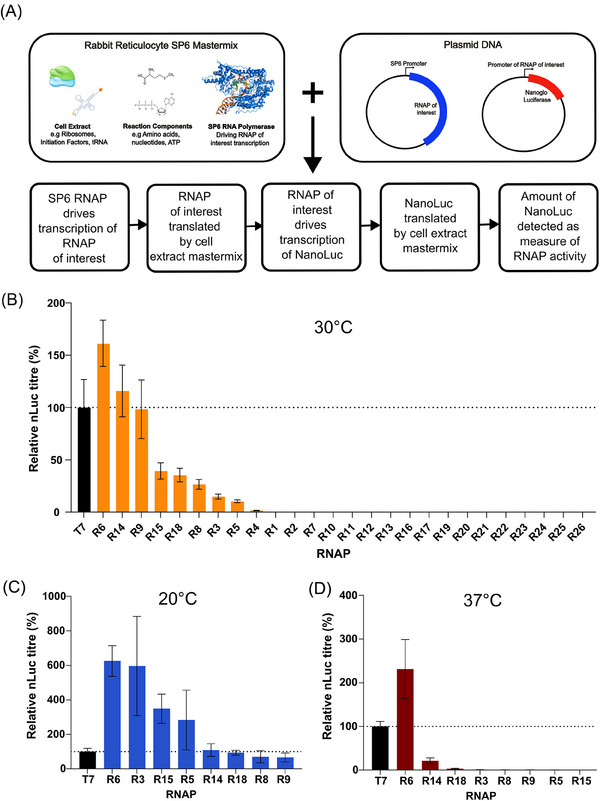
RNA polymerases (RNAPs) were functionally characterized in a cell‐free coupled transcription‐translation system (A). Protein coding and cognate promoter sequence pairs were inserted into screening platform vectors and incubated with SP6‐rabbit reticulocyte mastermix at 30°C (B), 20°C (C), and 37°C (D). Luciferase expression was quantified 1 h post‐incubation; data are expressed as a percentage of the production achieved using the control T7 RNAP. Values represent the mean + standard deviation (SD) of three independent experiments (*n*  =  3, each performed in triplicate).

Previous studies focused on identifying new RNAP biocatalysts for mRNA manufacture have relied on recombinant production of individual “test” enzymes in *E. coli* cell‐hosts, prior to characterization in IVT reactions.^[^ ^18^, ^19^, ^30^ ^]^ This time‐consuming method is undesirable for characterization of a large RNAP library, particularly given that manufacture of complex proteins at appropriate yield and quality can require significant process optimization.^[^ ^31^, ^32^ ^]^ Moreover, variation in recombinant protein stability and purity may prevent accurate quantification of relative enzyme activities across the library. Accordingly, to functionally characterize our 26 novel RNAPs in parallel, we developed a high‐throughput testing platform that does not require production and purification of each polymerase. This was achieved by adapting a cell‐free coupled transcription‐translation system that has previously been employed to rapidly assess activity of variant T7 polymerases.^[^ ^33^, ^34^ ^]^ As shown in Figure 2A, this platform utilizes a mastermix containing rabbit reticulocyte lysate and recombinant SP6 RNAP to facilitate in vitro production of a “test” RNAP, which then in turn drives expression of a Nano‐luciferase reporter‐gene under the control of its cognate promoter.

Protein coding sequences and predicted cognate promoter elements for each of the 26 test enzymes were chemically synthesized and inserted into the appropriate RNAP screening platform vectors (Figure 2A). Resulting plasmid‐pairs were individually mixed with the SP6 RNAP‐rabbit reticulocyte lysate mastermix, and luciferase production was measured after incubating the reaction for 1 h at 30°C (recommended assay reaction temperature). As shown in Figure 2B, 8/26 enzymes were functionally active, driving luciferase expression levels that ranged from 10% to 161% of that achieved using the control T7 RNAP. Accordingly, ≈70% of tested enzymes were non‐functional, highlighting the difficulty associated with identifying novel RNAP biocatalysts.

There was no significant correlation between predicted enzyme temperature optima and observed activity at 30°C. However, to further assess the impact of reaction parameters on polymerase performance, we tested enzyme activities at increased (37°C) and decreased (20°C) temperatures. While the same eight RNAPs were functional at 20°C, only three of these enzymes displayed activity at 37°C. Moreover, apart from R6 which drove highest luciferase expression levels under all conditions tested, the relative performance of polymerases varied with temperature. Although our objective was to identify effective polymerase‐promoter pairs, rather than to precisely elucidate their relative performance characteristics, these data indicate that enzymes active over a narrow range of temperatures may be incorrectly categorized as non‐functional. However, we concluded that this was unlikely when testing across three separate temperature set‐points, and that enzyme inactivity in our screening platform was more likely due to either (i) inaccurate annotation/sequencing of putative RNAP coding sequences or (ii) incorrect promoter prediction.

Although enzyme activity in the cell‐free screening system may not be directly predictive of performance in IVT‐based mRNA manufacturing processes, it is notable that polymerase R6 drove higher levels of luciferase expression than T7 in all conditions tested (increase ranging between 160% and 620%), including a 220% increase at T7s optimum reaction temperature (37°C). Five further RNAPs (R3, R5, R9, R14, R15) facilitated luciferase titers greater than or equal to that achieved with T7 in at least 1 reaction condition. Accordingly, these enzymes may exhibit higher processivity/catalytic activity than T7 and could therefore have potential use in enhancing mRNA production yields. Moreover, their use may permit simplified downstream processing operations via reduced formation of product‐related impurities, particularly as many of these RNAPs exhibit relatively high activities at low temperatures.^[^ ^30^ ^]^

**FIGURE 3:**
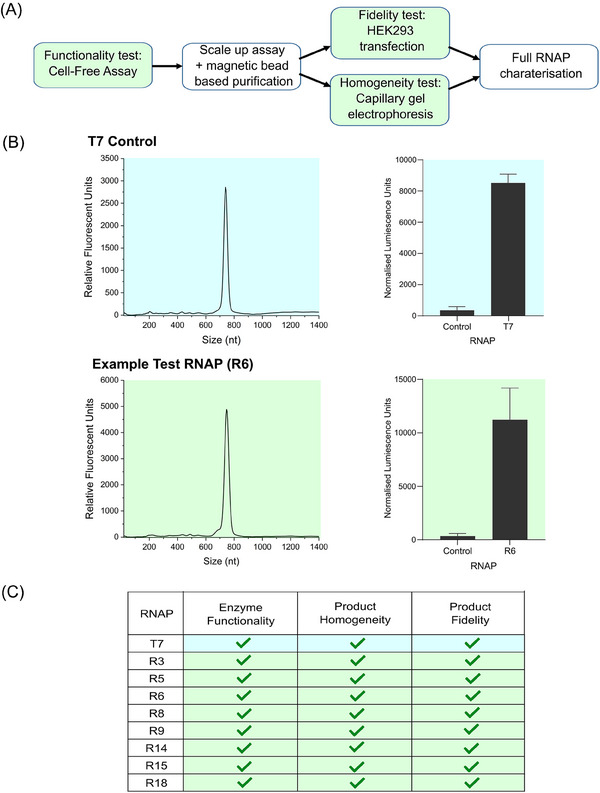
(A) Process flow diagram for high‐throughput assessment of novel RNA polymerases (RNAPs); enzymes must pass key performance tests (shown as green boxes) to progress to the next analysis step. (B) Functional RNAPs (Figure 2) were used in scaled‐up cell‐free coupled transcription‐translation assays to produce luciferase mRNA that was subsequently purified using Oligo‐(dT) magnetic beads. mRNA quality was profiled by (i) capillary gel electrophoresis analysis and (ii) transfection into HEK293 cells prior to measurement of luciferase protein levels after 48 h (values represent the mean + standard deviation [SD] of three independent experiments). Electropherogram peak distribution and luciferase enzyme activity units were compared to benchmark T7 controls (shown in blue) to confirm whether test RNAPs (example profile of R6 shown in green) had passed product homogeneity (presence of similar single sharp peak) and fidelity (measurable luciferase activity) assessments. (C) Quality analytics profile for all functional RNAPs identified in this study. Enzymes which pass all three performance tests are considered promising candidates for use in in vitro transcription (IVT)‐based mRNA production processes.

Beyond raw catalytic power, that is, the relative rate at which each an enzyme produces mRNA from a given mass of DNA template (Figure 2), there are several other properties that would ideally be characterized by an in vitro RNAP screening platform. A complete comparative assessment would include measurement of a wide range of impurities, however current methodologies to quantify critical attributes such as dsRNA levels and polyA tail homogeneity are incompatible with a high‐throughput small‐scale testing module. While a comprehensive quantitative analysis is therefore intractable, we reasoned that the utility of the platform would be significantly enhanced by developing a standardized secondary product‐quality test to further evaluate RNAPs that pass the initial “functionality assessment”. As shown in Figure 3A, using the eight identified functional enzymes, we performed scaled‐up (80 µL) cell‐free coupled transcription‐translation assays, before Oligo‐(dT) based magnetic bead affinity enrichment of resulting luciferase mRNA molecules. Purified product samples were split into two downstream quality assessment tests to evaluate (i) generation of product‐related impurities (i.e., proportion of full‐length mRNA) by CGE analysis and (ii) sequence fidelity/functionality by transfection into HEK 293 cells followed by quantification of Luciferase protein levels. The resulting data profiles were then compared to the T7 control, where enzymes that produced a single sharp peak in the electropherogram, and measurable active luciferase in the cell‐based assay were designated as passing the quality evaluation step (Figure 3B). These data confirmed that all eight enzymes produced functional homogenous mRNA molecules (Figure 3C), validating the use of the quality assessment tests and confirming the potential utility of these RNAPs. While further complete characterization is required to fully assess their bioindustrial application, the identification of eight novel functional enzymes more than doubles the number of available RNAPs, expanding the biocatalyst solution space for mRNA manufacture by ≈130% (i.e., an expansion from 6 to 14 total available enzymes).

### 3.3 Cognate promoter prediction is the critical limiting factor restricting further expansion of the RNAP biocatalyst solution space

**FIGURE 4:**
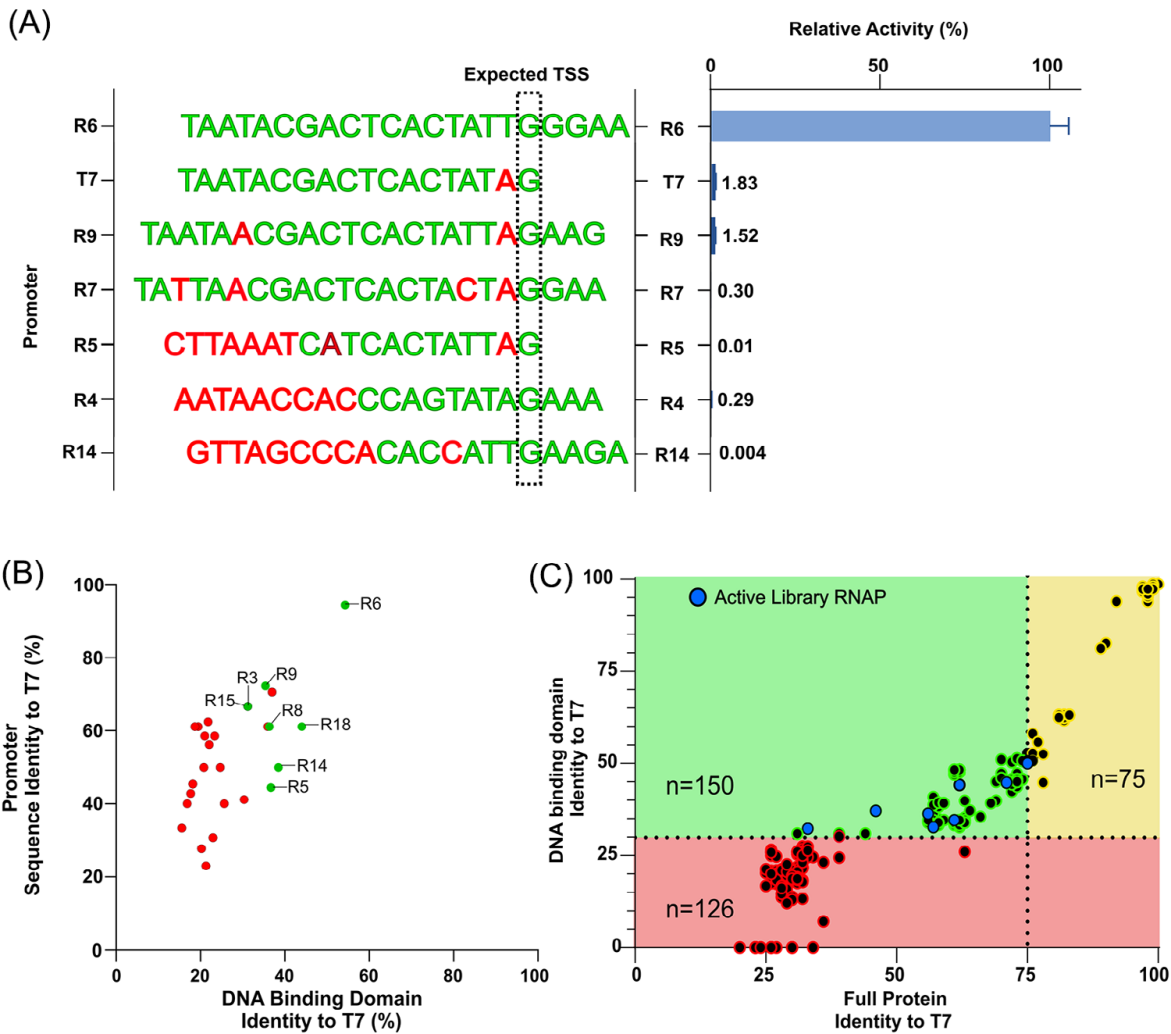
(A) The ability of RNA polymerase (RNAP)6 (*Pectobacterium* phage PP74) to drive Luciferase expression from varying non‐cognate promoter elements was evaluated in cell‐free coupled transcription‐translation assays (see Figure 2). Data are expressed as a percentage of the production achieved using the cognate RNAP‐6 promoter. Values represent the mean + SD of three independent experiments (*n*  =  3, each performed in triplicate). (B) Test library RNAP‐promoter pairs were analyzed to determine relative DNA binding domain and promoter sequence identity with T7. Pairs that were found to be active or inactive in functional characterization tests are shown as green and red dots respectively. (C) The entire theoretical RNAP biocatalyst solution space was analyzed to identify promising future targets for in vitro characterization (green section). Enzymes predicted to have incorrect promoter definitions or similar bioproduction phenotypes to T7 are shown in the red and yellow sections respectively. Functional RNAPs identified in this study are shown as blue dots.

The finding that ≈70% of characterized enzymes were non‐functional in in vitro tests (Figure 2) indicates that the vast majority of putative RNAPs cannot be simply extracted from online databases and directly employed in mRNA manufacturing applications. Given that RNAPs are known to display highly stringent promoter recognition requirements,^[^ ^26^ ^]^ we hypothesized that enzyme inactivity may have resulted from inaccurate predictions of cognate promoter sequences. To exemplify this, we characterized the ability of R6, the best performing polymerase in in vitro screens, to initiate transcription from the promoters of other functional enzymes. As shown in Figure 4A, R6 could not drive quantifiable gene expression from any of these variant elements, where even a single nucleotide change was sufficient to completely abolish transcriptional output. These data highlight that the ability to exploit any given potential RNAP biocatalyst is heavily dependent on highly accurate definition of its cognate promoter sequence.

We reasoned that RNAP promoter prediction tools may be incapable of precisely defining new elements that are significantly divergent from currently known sequences, as evidenced by our inability to derive cognate promoters for ≈40% of bioinformatically determined RNAP clusters (see Section 3.1). Existing promoter prediction algorithms are primarily trained based on known promoter sequences, and their predictive ability is accordingly limited for novel test enzymes with significantly different sequences and structures.^[^ ^24^, ^35^ ^]^ Indeed, given the paucity of characterized RNAP promoters, novel “test” enzymes may recognize sequence motifs and architectures that are (i) substantially different to those used to train/design current algorithms, and accordingly (ii) beyond the predictive capabilities of available tools. Rationalizing that divergence in promoter structure/sequence would be underpinned by differences at the amino acid level, we investigated whether enzyme inactivity was associated with DNA binding domain sequences that varied significantly to those of well‐studied biocatalysts. As shown in Figure 4B, 8/10 polymerases that share relatively high DNA binding domain sequence identity with T7 (>30%) were found to be active, while all 16 enzymes that share relatively low similarity (<30%) were non‐functional. This indicates that accurate prediction and assessment of new RNAPs may not be possible when DNA binding domain sequence similarity to well‐studied enzymes falls below a certain threshold. In contrast, cognate promoter sequence similarity with T7 promoter was not a good predictor of RNAP functionality, where 6/8 active and 12/16 inactive elements shared between 40% and 65% sequence identity with T7 (Figure 2B). We therefore concluded that comparative amino acid sequence analysis with T7 is currently the most effective method to refine libraries of putative RNAPs by identifying enzymes that are unlikely to be functional in in vitro testing.

Our findings indicate that potential RNAPs can be efficiently screened in silico, where enzymes that share below ≈30% DNA‐binding domain sequence identity with T7 are unlikely to be functional in vitro owing to incorrect promoter definition. However, as shown in Figure 4C, this cut‐off removes ≈36% of the theoretical biocatalyst solution space for mRNA production. Of the remaining 225 polymerases, 75 share relatively high overall protein sequence identity (>75%) with T7. Such enzymes are considered unlikely to exhibit substantial differences to T7 in key performance criteria such as enzyme processivity and product‐related impurity generation. Accordingly, only 150 RNAPs are predicted to be both active in vitro and potentially display novel, desirable bioproduction functionalities (including the eight we have identified in this study). This analysis therefore highlights 142 promising additional biocatalyst targets for future investigation, including 88 that do not share high sequence identity (> 75%) with either the six previously characterized RNAPs or the eight enzymes identified in this study (listed in Table S1). However, it also suggests that >120 potentially useful enzymes are currently difficult to exploit, highlighting promoter prediction capability as the key limiting factor preventing comprehensive exploitation of the theoretical biocatalyst solution space for mRNA production. Although the cognate promoters of individual polymerases can be elucidated via non‐bioinformatic laboratory techniques,^[^ ^18^ ^]^ these time‐intensive methods are intractable when testing multiple enzymes in parallel. Accordingly, full exploration of the putative RNAP biocatalyst solution space to optimize mRNA production processes will likely require significant advancements in phage promoter prediction tools.

### 3.4 Novel identified RNAPs enhance the biocatalyst solution space for IVT‐based mRNA production

**FIGURE 5:**
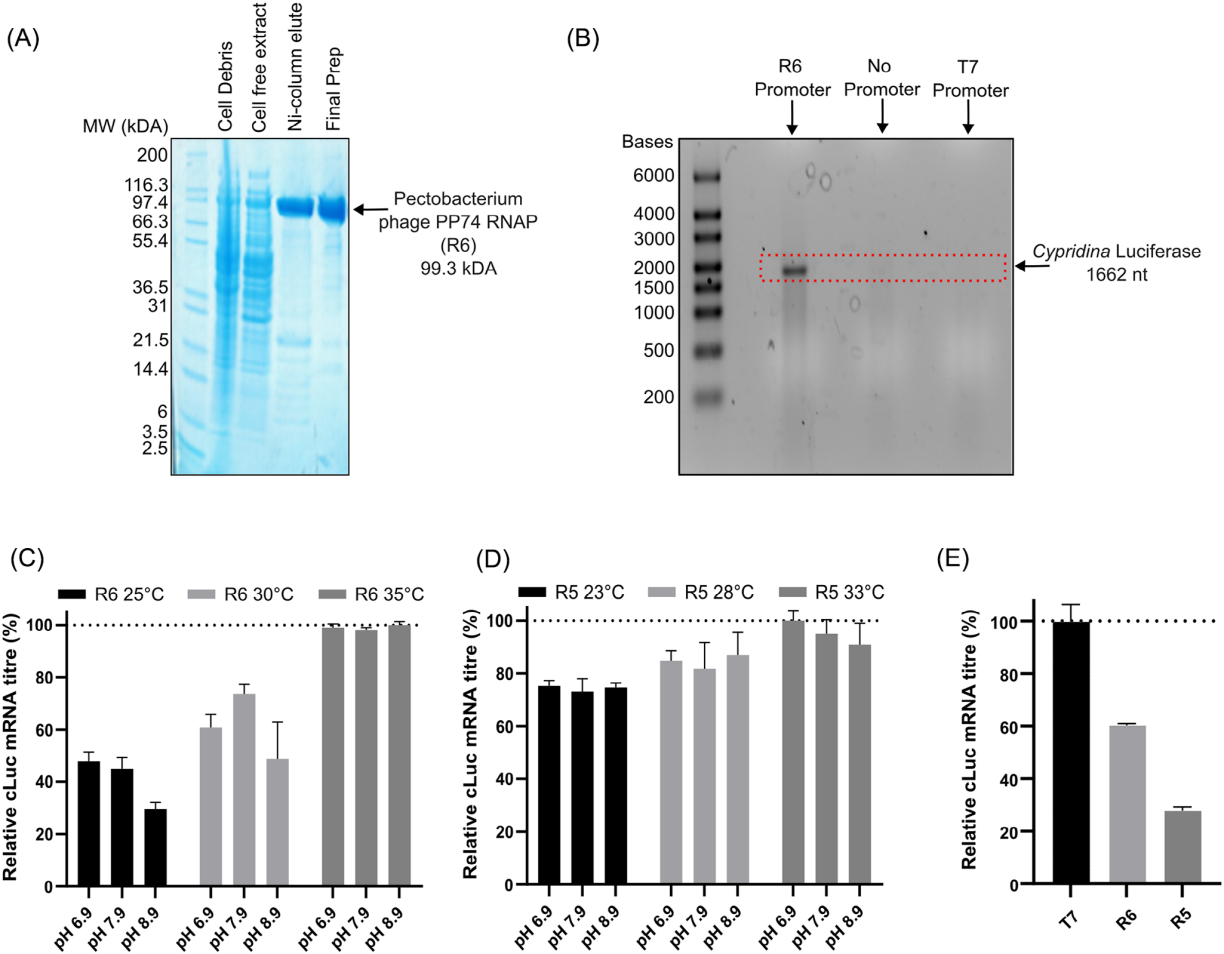
RNA polymerase (RNAP) 5 and 6 were recombinantly produced in *E. coli* and purified using His‐tag affinity and size exclusion chromatography (A). Purified enzymes were used to manufacture luciferase mRNA in in vitro transcription (IVT) reactions, and production of full‐length product was verified by gel electrophoresis analysis (B). IVT production processes utilizing RNAP6 (C) and RNAP5 (D) were performed in varying reaction conditions and resulting mRNA titers were quantified using nanodrop spectrophotometry. Using identified optimal reaction parameters for each enzyme, the relative performance of recombinant polymerases was evaluated compared to an NEB T7 control (E). Data in C and D are expressed as a percentage of the production achieved using optimal reaction parameters for each enzyme. Data in (E) are expressed as a percentage of the production achieved using T7. In (C), (D), and (E) values represent the mean +  standard deviation (SD) of three independent experiments (*n*  =  3, each performed in triplicate).

To validate that novel RNAPs identified via our HT cell‐free screening platform have utility in mRNA manufacturing processes, we recombinantly produced polymerases R5 and R6 in *E. coli*. These polymerases were chosen to represent highly and moderately active enzymes, where R6 (*Pectobacterium* phage PP74) was previously shown to be the best performing RNAP in all temperatures tested, and R5 (*Dickeya* phage Mysterion) drove relatively low‐to‐medium levels of transcription across varying reaction conditions (Figure 2). Polymerases were overexpressed in 0.5 L scale production processes and purified using His‐tag affinity and size exclusion chromatographic operations. Purified recombinant RNAPs were then utilized in IVT reactions to manufacture *Cypridina* luciferase (CLuc) mRNA. As shown in Figure 5, both enzymes drove significant levels of Cluc expression, validating their function as biocatalysts for synthetic mRNA production. To evaluate enzyme robustness, we tested the performance of each RNAP at a range of pH (predicted optimum ± 1) and temperature (predicted optimum ± 5°C) set‐points. Both RNAPs were functional across all conditions tested, where R5 performance was relatively constant, and R6 activity increased with temperature. The latter highlights that expected phage host growth temperatures are not directly predictive of optimal in vitro reaction conditions for recombinant RNAPs.

As shown in Figure 5, utilization of R6 at “optimal” reaction parameters (pH 7.9, 35°C) facilitated mRNA product titers >60% of that achieved when using NEB recombinant T7 at recommended conditions (pH 7.9, 37°C). Although R6 drove higher levels of gene transcription than T7 in our cell‐free system, it is not surprising that T7s relative activity was enhanced in IVT processes given that NEB T7 is a highly pure engineered enzyme with fully optimized reaction conditions. Indeed, we anticipate that rational protein engineering/evolution, coupled with improved purification techniques and reaction parameters (e.g., optimized MgCl_2_ concentration), will significantly increase R6s biocatalytic activity in IVT‐based mRNA production. Irrespective of this, by initially facilitating product yields equivalent to ≈61% of that achieved by optimized T7, R6 is considered a highly active biocatalyst.

Polymerase R5 enabled product titers ≈47% of that achieved using R6, suggesting that the comparative performance of novel RNAPs in cell‐free testing platforms is broadly predictive of their relative ability to maximize mRNA yields in IVT manufacturing processes. Accordingly, we concluded that the additional six novel enzymes identified in this study (*Erwinia amylovora* phage Era103, *Pectobacterium* phage DUPP II, *Pectobacterium* phage Jarilo, *Delftia* phage IME‐DE1, *Pseudomonas* phage Henninger, and *Citrobacter* phage CR8) are also likely to facilitate moderate‐to‐high mRNA production yields. While we cannot currently comment on the relative ability of these new polymerase to enhance product quality, previous work suggests they will generate variable levels of product‐related impurities, such as dsRNA and truncated species.^[^ ^18^, ^19^, ^20^, ^30^, ^36^ ^]^ Indeed, this new library is particularly likely to exhibit differential bioproduction phenotypes, given that they were specifically selected based on sharing minimal amino acid sequence similarities. We therefore conclude that addition of these eight novel functional enzymes to the RNAP biocatalyst solution space will significantly enhance IVT‐based mRNA manufacturing optimization strategies. We note that industrialization of these enzymes will be dependent on additional factors, particularly the ability to manufacture them at high yields in large‐scale production processes and their relative long‐term stability.^[^ ^31^ ^]^ While the two RNAPs tested here could be produced using standard upstream and downstream unit operations, full validation studies for all new enzymes will need to include comprehensive manufacturability assessments.

## 4 CONCLUDING REMARKS

The eight novel sequence‐diverse functional RNAPs identified in this study substantially increases the number of biocatalysts available for mRNA production. Although further work is required to comprehensively define their relative performance characteristics, particularly their associated impurity generation profiles, this expansion of the biocatalyst solution space significantly enhances design options for molecule‐, process‐, and application‐specific optimization of mRNA product yield and quality. This improved flexibility will become increasingly useful as mRNA product lines continue to diversify toward large, complex molecules that pose new manufacturing challenges.^[^ ^11^, ^12^, ^14^, ^15^, ^16^ ^]^ Our combined in silico and in vitro analysis of the theoretical biocatalyst solution space (i) showed that full exploitation of potential RNAPs is restricted by cognate promoter prediction capabilities, but, also (ii) identified a panel of enzymes that are particularly promising for future investigation.

The screening platform we have developed can be utilized to rapidly test additional RNAP libraries. For example, this will be useful for responsive functional characterization of putative enzymes that are newly‐deposited in online protein databases each year. Moreover, it can be applied to significantly increase the throughput for testing rationally engineered enzyme variants. Protein engineering is a promising strategy to enhance the performance of candidate RNAPs, for example, by reducing the impurity generation profile of an enzyme that naturally exhibits high processivity rates. Our platform will permit initial functional screening of such libraries without requiring time‐intensive processes associated with manufacturing each RNAP individually. Further, it could be utilized for product‐RNAP matchmaking, to align mRNA molecule‐specific features (e.g., length, GC content, secondary structure) with enzyme‐specific performance characteristics. This may be particularly applicable to development of small‐batch customized mRNA therapeutics that require cost‐effective methods to optimize production of novel sequences.^[^ ^1^, ^37^ ^]^ However, to fully harness it's potential for such applications the screening system will require further development, particularly the creation of additional product quality analytics modules that can quantify critical impurity levels such as dsRNA.^[^ ^38^ ^]^ In conclusion, by more than doubling the number of available polymerases, and providing associated methods to select and screen further new enzymes, this study has facilitated a significant expansion of the RNAP biocatalyst solution space, enhancing strategies to optimize and troubleshoot IVT‐based mRNA production processes.

## AUTHOR CONTRIBUTIONS

**Edward Curry**: Conceptualization; methodology; analysis; investigation; manuscript preparation. **Svetlana Sedelnikova**: Investigation; analysis. **John Rafferty**: Investigation; analysis. **Martyn Hulley**: Conceptualization; analysis. **Melinda Pohle**: Investigation; analysis. **George Muir**: Investigation; analysis. **Adam Brown**: Conceptualization; methodology; analysis; manuscript preparation; funding acquisition.

## CONFLICT OF INTEREST STATEMENT

The authors declare no conflict of interest.

## Supporting information

Supporting information

## Data Availability

The data that support the findings of this study are available from the corresponding author upon reasonable request.
